# *H. capsulatum*: A Not-So-Benign Cause of Pericarditis

**DOI:** 10.1155/2017/3626917

**Published:** 2017-09-07

**Authors:** Paolo K. Soriano, Muhammad Iqbal, Shakthishri Kandaswamy, Sami Akram, Abhishek Kulkarni, Tamer Hudali

**Affiliations:** ^1^Department of Internal Medicine, Southern Illinois University, 751 N Rutledge, Springfield, IL 62702, USA; ^2^Infectious Disease, Southern Illinois University, 751 N Rutledge, Springfield, IL 62702, USA; ^3^Division of Cardiology, Southern Illinois University, 751 N Rutledge, Springfield, IL 62702, USA; ^4^Clinical Internal Medicine, Southern Illinois University, 751 N Rutledge, Springfield, IL 62702, USA

## Abstract

The common causes of pericarditis and its course are benign in the majority of cases. Thus, further testing is usually not pursued and treatment for a presumptive viral etiology with nonsteroidal agents and steroids has been an accepted strategy. We present a patient with pericarditis who was unresponsive to first-line therapy and was subsequently found to have necrotizing granulomas of the pericardium with extensive adhesions and fungal elements seen on tissue biopsy. Serologic testing confirms active* H. capsulatum* infection, and he responded well to Itraconazole treatment. In patients with pericarditis who fail standard therapy with NSAIDs and steroids, it is suggested that they undergo thorough evaluation and that histoplasmosis be considered as an etiology, especially in endemic regions.

## 1. Case Introduction

We report an uncommon case of an adult male from the Midwestern United States presenting with as acute pericarditis due to* Histoplasma capsulatum* infection. We reviewed the literature on* H. capsulatum* pericarditis and discussed the diagnosis and management of necrotizing granulomatous pericarditis due to* H. capsulatum*. We recommend that histoplasmosis infection be on the differential diagnoses in patients having acute pericarditis in endemic regions.

## 2. Case History

A 56-year-old male with obesity, hypertension, diabetes mellitus type 2, and dyslipidemia presented with nonexertional chest pain that wakes him up at night exacerbated by coughing and laying supine. There were no associated fever, dizziness syncope, and palpitation. He recalls symptoms of an upper respiratory tract infection which have resolved a few weeks earlier. Our patient is an office worker with no additional risk factors as smoking, chest trauma, recent travel, significant outdoor exposure, and family history of malignancy.

On evaluation, blood pressure was 144/68 mmhg, HR was 101 bpm, RR was 24 cpm, and the patient was afebrile. Eye and oral examination showed no suspicious lesions. There was no skin rash and synovitis. Cardiovascular examination was negative for jugular venous distention and hepatojugular reflex. Precordium was mildly tender, point of maximal impulse was adynamic and nondisplaced, and there was no palpable ventricular heave. On auscultation, S1 and S2 sounds were muffled; a precordial rub was appreciated over the left sternal border and is heard throughout the cardiac cycle. The remainder of the physical examination was unremarkable.

Electrocardiography showed nonspecific ST-T wave changes and no PR abnormalities. Troponin values were normal. A transthoracic echocardiogram demonstrated a mildly thickened pericardium with no pericardial effusion. Global ventricular wall motion was normal. Results of the initial laboratory investigation are outlined in Table 1. This shows leukocytosis with lymphopenia and monocytosis, elevated ESR, and normal renal function. The patient was then started on a course of Ibuprofen and Prednisone for suspected viral pericarditis.

**Figure 1:**
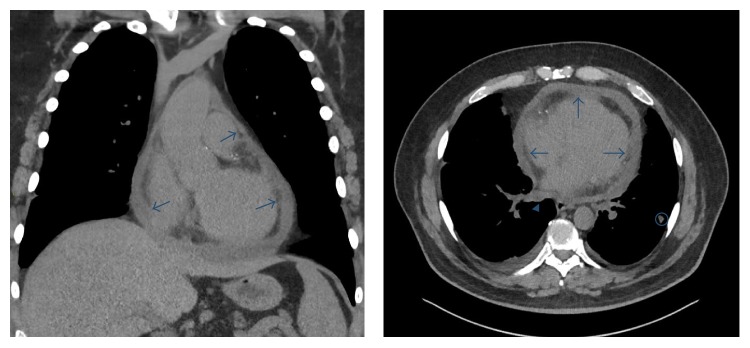
Chest CT imaging. Coronal view of noncontrast chest CT scan showing hyperattenuated pericardium (arrows). Axial chest CT scan showing the same diffusely thickened pericardium. Enlarged mediastinal lymph node (arrowhead). Note the solitary nodule in the Left lower lobe (encircled).

During his 4-week follow-up, the patient reported some improvement of the chest discomfort but now endorses decreased exercise tolerance, exertional dyspnea, and worsening fatigue. D-Dimer was obtained and was slightly elevated, 0.47 (reference: 0.27–0.40 mcg/ml). Computed tomography of the chest demonstrated no pulmonary artery embolism. The pericardium was markedly thickened, along with a small amount of nonloculated pericardial effusion. Visualized were multiple enlarged mediastinal and subcarinal lymph nodes and a 1.2 cm pulmonary nodule in the left lower lobe (Figure 1). Taking his risk factors into account, his symptoms were now concerning for coronary ischemia. Myocardial Perfusion Scanning revealed a moderately intense area of reversible defect consistent with ischemia and infarction of the inferior wall. There were no features of hemodynamic collapse. Subsequent Coronary Angiography confirmed severe three-vessel coronary artery disease* (mid-LAD 80%, 1st Diagonal 90% ostial lesion, 2nd Diagonal 80%, and RCA 100% with collaterals)*. Left ventriculogram showed hypokinesis of the basal inferior myocardium with an estimated EF of 50%.

**Figure 2:**
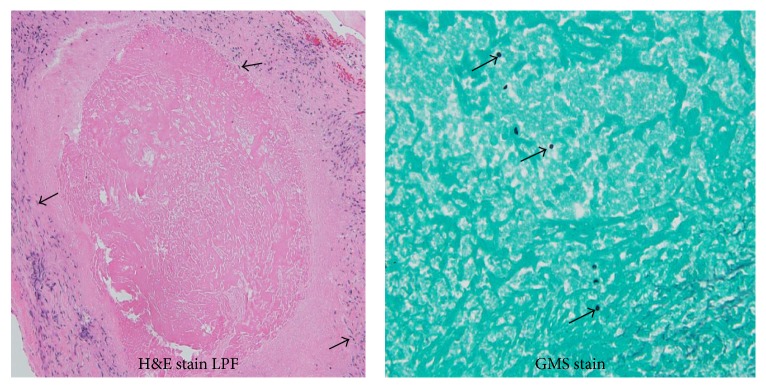
Histologic images. H&E stain of the atrial appendage showing epithelioid histiocytes (arrows), lymphocytes, and caseating granuloma (high power view of hematoxylin-eosin stain). Fungal elements (arrows) within central necrosis (Grocott methenamine silver stain, 400x magnification).

The patient underwent urgent Coronary Artery Bypass Grafting (CABG). Intraoperatively, upon violating the pericardial sac, it was immediately evident that he had extensive nodular adhesions on the visceral and parietal pericardium. Gram stain and cultures for bacteria and acid fast bacilli were negative. Histopathology demonstrated a necrotizing granulomatous inflammation that stained positive for fungal elements within the central necrotic tissue. No malignant cells were identified (Figure 2). Screening for sarcoidosis, tuberculosis, and HIV infection was negative. Further serologic testing confirms* H. capsulatum* infection (Table 1). His postoperative course remained unremarkable. Treatment with Itraconazole was initiated with instructions to complete a 6-month course and a follow-up chest CT scan to monitor progression of the visualized solitary pulmonary nodule.

## 3. Discussion

Our index case fulfilled the clinical diagnostic criteria for acute pericarditis with typical chest pain, pericardial friction rub, and new pericardial effusion [1]. There were no diffuse ST-T wave changes on electrocardiogram and there was no pericardial effusion on echocardiography. The diagnosis is clinical and, in eighty percent of cases, the etiology is viral [2–4]. Therapy is empirically directed towards inflammation [3, 5].

Management of his condition included prednisone and nonsteroidal anti-inflammatory agents. Due to the benign course of the illness associated with the common causes of pericarditis, it is not necessary to search for the etiology in all patients [2–4], especially in areas with low rates of tuberculosis [6]. Hence, treatment for a presumptive viral etiology is an acceptable strategy. Even with extensive evaluation, a definite cause is established in only 16% of cases [7, 8]. Similarly, in a three case series that included a total of 784 unselected patients who underwent extensive testing, a specific diagnosis was only obtained in 130 cases (17%) [7, 9, 10].

*H. capsulatum* is a thermally dimorphic fungus endemic to North and Central America and is a common cause of fungal pericarditis in patients with an intact immune system [11–13].* Histoplasma* pericarditis was first described in 1955 by Billings and Couch in a patient with pericardial calcification and positive histoplasmin skin test [14]. Three years later, Heiner (1958) reported a case of constrictive pericarditis in a pediatric patient. The etiologic agent was diagnosed as* H. capsulatum* by skin and serologic testing [15]. Perhaps the largest case series, with sixteen patients, by Picardi et al. (1976) concluded that this form of granulomatous pericarditis carries a good prognosis [16]. To date, we found approximately 35 reported cases of pericarditis caused by* H. capsulatum*.

The pathogenesis of pericarditis due to histoplasmosis is thought to be due to hypersensitivity to antigens from the yeast-phase cells of* H. capsulatum* within the mediastinal lymph nodes or adjacent pulmonary focus of the host [16, 17]. Pericardial involvement occurs as a complication of inflammation and represents an inflammatory condition rather than infection of the pericardium [18]. This explains why organisms are not recovered from pericardial fluid and cannot typically be isolated and cultured from biopsy specimens [19, 20].

In biopsy specimens, granulomas are common [13, 21]. Granulomas are a form of highly effective, nonspecific antifungal immune response mediated by macrophages infected with* Histoplasma*. This mechanism is essential to contain fungal growth, prevent systemic dissemination, and protect the organs from widespread inflammatory tissue damage [22]. The cytokines involved in this process are mainly TNF-*α* and IFN-*γ* [22]. This places histoplasmosis in the differential diagnosis of sarcoidosis, tuberculosis, and malignancy. It is especially important that the distinction between sarcoidosis and histoplasmosis be made. Failure to exclude the diagnosis of histoplasmosis before treatment for a cardiac sarcoid may lead to marked disease exacerbation if immunosuppressive therapy is initiated in a patient who actually has acute histoplasmosis [23]. Ineffective cell-mediated immunity leads to the inability to control reticuloendothelial spread of histoplasmosis and disseminated disease [13, 24, 25]. In this case, the angiotensin-converting enzyme level and the QuantiFERON-TB test were notably negative. Suspicion for fungal etiology was triggered when fungal elements were visualized on GMS staining. The diagnosis of histoplasmosis was eventually made with serologic testing (Table 2).

A variety of tests may be utilized for detecting histoplasmosis including histopathology stains for fungi, fluid, and tissue cultures and Ag-Ab detection. Complement fixation and immunodiffusion (IF) are both serologic tests utilized in* H. capsulatum* detection with similar sensitivities above 90% [26]. The immunodiffusion test is less sensitive compared to immunodiffusion but has specificity of almost 100% [27, 28] In immunodiffusion, test results are reported as M or H precipitins or bands. Most patients will develop an M band, while the H precipitin band is detectable in fewer than 20% of cases and is seen most often in patients with disseminated disease [29]. Antigen testing in our patient revealed comp-fix titers of 1 : 8 and a positive M band with IF. Although higher comp-fix titers are highly suggestive of an acute infection, lower titers as seen in this case are seen in one-third of active disease.

Management of* Histoplasma* pericarditis is based on the guideline released in 2007 by the Infectious Diseases Society of America. NSAIDs are the mainstay of therapy for mild disease [18]. In the event of hemodynamic compromise and in individuals who fail to improve on NSAIDs, prednisone may be used. Pericardiocentesis is also indicated in those with evidence of hemodynamic compromise. Although there is no active pericardial fungal infection, the use of Itraconazole is advocated in moderate-to-severe disease [18]. However, due to lack of clinical trials, it is unclear whether antifungal therapy alters the course of histoplasmosis pericarditis [30].

Once treatment is initiated, a quantitative antigen enzyme immunoassay may be used to monitor decreases in serum and urine* H. capsulatum* antigens [31]. This strategy was not employed for our patient, since he had very low antigen titers before treatment. In this situation, there is no test to document disease eradication. Improvement is assessed based solely on clinical findings.

Since the submission of this article, our patient presented for biopsy of the left lower lobe pulmonary nodule. Results reveal a benign tissue with necrotizing granuloma, negative for fungal and mycobacterial elements. No repeat antigen testing was performed and he remains asymptomatic to this day.

## 4. Conclusion

We present a rare case of histoplasmosis pericarditis that failed therapy with NSAIDs and steroids but responded to Itraconazole treatment. We recommend that, in endemic regions, clinicians should further evaluate patients with typical symptoms of pericarditis and consider histoplasmosis as an etiology, especially in patients that fail first-line therapy.

## Figures and Tables

**Table 1:** Laboratory evaluation and serologic testing.

WBC	7.4 (3.4–9.4 K/mm^3^)
Neut	64% (47–67%)
Lymph	21% (25–45%)
Mono	12% (1–9%)
Eos	2% (0–6%)
ESR	25 (0–20 mm/hr)
Blood cultures	Negative
Serum calcium	9 (8.5–10.5 mg/dl)
BUN	14 (6–22 mg/dl)
Serum creatinine	0.9 (0.7–1.4 mg/dl)
*Histoplasma* yeast Ab	<1 : 8 (normal < 1 : 8)
*Histoplasma* mycelial Ab	<1 : 8 (normal < 1 : 8)
*Histoplasma IF*	*Positive (M band)*
*Blastomyces* Ab	<1 : 8 (normal < 1 : 8)
*Blastomyces* IF	Negative
*Coccidioides* Ab	<1 : 2 (<1 : 2)
*Coccidioides* IF	Negative
*Aspergillus* IF	Negative
Angiotensin-converting enzyme	12 (9–67)
HIV 1 & 2 Ab	Nonreactive
QuantiFERON-TB	Negative

ESR: erythrocyte sedimentation rate; BUN: blood urea nitrogen; Ab: antibody; IF: immunodiffusion.

**Table 2:** Etiology of acute pericarditis: data from 3 large case series.

	Permanyer-Miralda et al. (*n* = 231)	Zayas et al. (*n* = 100)	Imazio et al. (*n* = 453)
Years	1977–1983	1991–1993	1996–2004
Location	Spain	Spain	Italy
Idiopathic	199	78	377
Specific etiology	32 (14%)	22 (22%)	76 (16.8%)
Neoplastic	12	7	23
Tuberculosis	9	4	17
Autoimmune	4	3	33
Purulent	2	1	3

Data from [7–9].

## References

[1] LeWinter M. (2005). *Pericardial diseases*.

[2] Troughton R. W., Asher C. R., Klein A. L. (2004). Pericarditis. *The Lancet*.

[3] Imazio M., Trinchero R. (2004). Clinical management of acute pericardial disease: a review of results and outcomes. *Ital Heart J*.

[4] Imazio M., Demichelis B., Parrini I. (2004). Day-hospital treatment of acute pericarditis: a management program for outpatient therapy. *Journal of the American College of Cardiology*.

[5] Imazio M., Adler Y. (2013). Treatment with aspirin, NSAID, corticosteroids, and colchicine in acute and recurrent pericarditis. *Heart Failure Reviews*.

[6] Adler Y., Charron P., Imazio M. (2015). ESC guidelines for the diagnosis and management of pericardial diseases: the task force for the diagnosis and management of pericardial diseases of the european society of cardiology(ESC) endorsed by: the european association for cardio-thoracic surgery (EACTS). *European Heart Journal*.

[7] Permanyer-Miralda G., Sagristá-Sauleda J., Soler-Soler J. (1985). Primary acute pericardial disease: a prospective series of 231 consecutive patients. *The American Journal of Cardiology*.

[8] Zayas R., Anguita M., Torres F. (1995). Incidence of specific etiology and role of methods for specific etiologic diagnosis of primary acute pericarditis. *The American Journal of Cardiology*.

[9] Imazio M., Cecchi E., Demichelis B. (2007). Indicators of poor prognosis of acute pericarditis. *Circulation*.

[10] Nemunaitis J., Ross M., Meisenberg B., O'Reilly R., Lilleby K., Buckner C. D. (1994;14(4):583-8). Phase I study of recombinant human interleukin-1 beta (rhIL-1 beta) in patients with bone marrow failure. *Bone Marrow Transplant*.

[11] Woods J. P., Heinecke E. L., Luecke J. W. (2001). Pathogenesis of *Histoplasma capsulatum*. *Seminars in Respiratory Infections*.

[12] Norman F. F., Martín‐Dávila P., Fortún J. (2009). Imported Histoplasmosis: Two Distinct Profiles in Travelers and Immigrants: Table 1. *Journal of Travel Medicine*.

[13] Kauffman C. A. (2007). Histoplasmosis: a clinical and laboratory update. *Clinical Microbiology Reviews*.

[14] Billings F. T., Couch O. A. (1955). Pericardial calcification and histoplasmin sensitivity. *Annals of Internal Medicine*.

[15] Heiner D. C. (1958). Diagnosis of histoplasmosis using precipitin reactions in agargel. *Pediatrics*.

[16] Picardi J. L., Kauffman C. A., Schwarz J., Holmes J. C., Phair J. P., Fowler N. O. (1976). Pericarditis caused by histoplasma capsulatum. *The American Journal of Cardiology*.

[17] Young E. J., Vainrub B., Musher D. M. (1978). Pericarditis Due to Histoplasmosis. *JAMA: The Journal of the American Medical Association*.

[18] Wheat L. J., Freifeld A. G., Kleiman M. B. (2007). Clinical practice guidelines for the management of patients with histoplasmosis: 2007 update by the Infectious Diseases Society of America. *Clinical Infectious Diseases*.

[19] Goodwin R. A., Nickell J. A., Des Prez R. M. (1972). Mediastinal fibrosis complicating healed primary histoplasmosis and tuberculosis. *Medicine*.

[20] Loyd J. E., Tillman B. F., Atkinson J. B., Des Prez R. M. (1988). Mediastinal fibrosis complicating histoplasmosis. *Medicine (Baltimore)*.

[21] Roberts W. C. (2005). Pericardial heart disease: its morphologic features and its causes. *Proc (Bayl Univ Med Cent)*.

[22] Heninger E., Hogan L. H., Karman J. (2006). Characterization of the Histoplasma capsulatum-Induced Granuloma. *The Journal of Immunology*.

[23] Sathapatayavongs B., Batteiger B. E., Wheat J., Slama T. G., Wass J. L. (1983). Clinical and laboratory features of disseminated histoplasmosis during two large urban outbreaks. *Medicine*.

[24] Kauffman C. A., Israel K. S., Smith J. W., White A. C., Schwarz J., Brooks G. F. (1978). Histoplasmosis in immunosuppressed patients. *The American Journal of Medicine*.

[25] Freifeld A. G., Iwen P. C., Lesiak B. L., Gilroy R. K., Stevens R. B., Kalil A. C. (2005). Histoplasmosis in solid organ transplant recipients at a large Midwestern university transplant center. *Transplant Infectious Disease*.

[26] Wheat J., French M. L. V., Kohler R. B. (1982). The diagnostic laboratory tests for histoplasmosis. Analysis of experience in a large urban outbreak. *Annals of Internal Medicine*.

[27] Bauman D. S., Smith C. D. (1976). Comparison of immunodiffusion and complement fixation tests in the diagnosis of histoplasmosis. *J Clin Microbiol*.

[28] Guimarães A. J., Nosanchuk J. D., Zancopé-Oliveira R. M. (2006). Diagnosis of histoplasmosis. *Brazilian Journal of Microbiology*.

[29] Picardi J. L., Kauffman C. A., Schwarz J., Phair J. P. (1976). Detection of precipitating antibodies to Histoplasma capsulatum by counterimmunoelectrophoresis. *The American Review of Respiratory Disease*.

[30] Wheat L. J., Smith E. J., Sathapatayavongs B. (1983). Histoplasmosis in Renal Allograft Recipients: Two Large Urban Outbreaks. *Archives of Internal Medicine*.

[31] Hage C. A., Kirsch E. J., Stump T. E. (2011). Histoplasma Antigen Clearance during Treatment of Histoplasmosis in Patients with AIDS Determined by a Quantitative Antigen Enzyme Immunoassay. *Clinical and Vaccine Immunology*.

